# Recent Progress in Solid-State Room Temperature Afterglow Based on Pure Organic Small Molecules

**DOI:** 10.3390/molecules29133236

**Published:** 2024-07-08

**Authors:** Xin Shen, Wanhua Wu, Cheng Yang

**Affiliations:** Key Laboratory of Green Chemistry & Technology of Ministry of Education, College of Chemistry, Sichuan University, 29 Wangjiang Road, Chengdu 610064, China

**Keywords:** organic room temperature afterglow, crystallization, molecular packing, host-guest doped, physical stimuli

## Abstract

Organic room temperature afterglow (ORTA) can be categorized into two key mechanisms: continuous thermally activated delayed fluorescence (TADF) and room-temperature phosphorescence (RTP), both of which involve a triplet excited state. However, triplet excited states are easily quenched by non-radiative transitions due to oxygen and molecular vibrations. Solid-phase systems provide a conducive environment for triplet excitons due to constrained molecular motion and limited oxygen permeation within closely packed molecules. The stimulated triplet state tends to release energy through radiative transitions. Despite numerous reports on RTP in solid-phase systems in recent years, the complexity of these systems precludes the formulation of a universal theory to elucidate the underlying principles. Several strategies for achieving ORTA luminescence in the solid phase have been developed, encompassing crystallization, polymer host-guest doping, and small molecule host-guest doping. Many of these systems exhibit luminescent responses to various physical stimuli, including light stimulation, mechanical stimuli, and solvent vapor exposure. The appearance of these intriguing luminescent phenomena in solid-phase systems underscores their significant potential applications in areas such as light sensing, biological imaging, and information security.

## 1. Introduction

**Figure 1:**
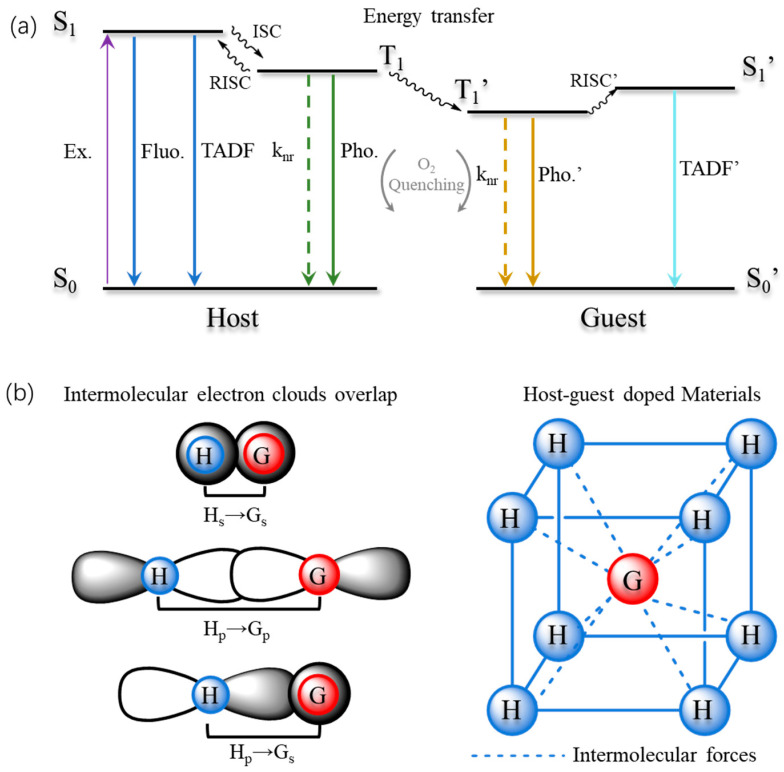
(**a**) Simplified Jablonski diagram for host-guest ORTA multiple luminescence; (**b**) intermolecular electron cloud overlap diagram and host-guest doped material interaction model.

Molecular interaction patterns significantly influence the luminescent behavior in solid-state systems, whether involving crystallized pure substances or doped systems. In solid-state systems, energy exchange predominantly occurs within approximately 10 Å, in accordance with the Dexter energy exchange principle [1,2,3]. When molecules are sufficiently close (~10 Å), their electron clouds overlap effectively, leading to an exponential increase in the exchange rate of the excited states with decreasing intermolecular distance (Figure 1b). The Simplified Jablonski diagram depicted in Figure 1a illustrates the transition processes of singlet and triplet excitons, alongside the intersystem crossing of triplet excitons between molecules. Following the transition of S_1_ to T_1_, the electron spins become oppositely oriented, rendering this process spin-forbidden and thereby resulting in a very low transition probability. The spin-orbit coupling (SOC) constant serves as a key parameter for quantifying this process. Theoretically, to achieve a high phosphorescence quantum yield (φ_phos_) in RTP materials (Equation (3)), at least three conditions must be satisfied: effective singlet-to-triplet intersystem crossing (increased SOC constant), adequate distribution of triplet excitons, rapid phosphorescence decay rate (τ_p_), and mitigation of non-radiative transitions (k_nr_) and T_1_ quenching (k_q_). The most crucial factor is enhancing the intersystem crossing efficiency (φ_ISC_), which Equation (3) identifies as setting an upper limit for φ_phos_. Accordingly, strategies involving heavy atom effects and supramolecular complexation [4,5,6,7,8,9,10,11,12,13] have been devised to enhance the SOC constant significantly, enabling the development of a range of organic small molecules capable of achieving room-temperature phosphorescence in solution. Nonetheless, the achievement of room-temperature phosphorescence in solution is hampered by generally localized excitons and collisional deactivation, necessitating relatively stringent conditions (e.g., oxygen-free environments), thereby restricting their practical applications.

^1^D* + ^1^A → ^1^D + ^1^A* (1)

^3^D* + ^1^A → ^1^D + ^3^A* (2)

(3) $$\varphi_{\mathrm{phos}\;}=\varphi_{\mathrm{ISC}}k_{p}\tau_{p}=\varphi_{\mathrm{ISC}}{(1-k_{q}-k}_{\mathrm{nr}})\tau_{p}$$

When two molecules are sufficiently proximate, triplet-state molecule A can transfer energy to ground-state molecule D via overlapping electron clouds, thereby inducing a transition of molecule D’s electrons to the triplet state. (Figure 1, Equation (2)). Theoretical considerations in solid-state luminescent systems suggest that a molecular distance of approximately 10 Å between the host and guest (Figure 1b) promotes significant overlap of their electron clouds. Concurrently, during the formation of solid-state donor-acceptor complexes, substances such as water and oxygen, which quench triplet-state excitation, are naturally excluded, establishing a dehydrated, deoxygenated, and motion-constrained environment. This reduction in the non-radiative transition rate (k_nr_) and triplet quenching rate by oxygen (k_q_) enhances the luminescence rate (k_p_), thereby favoring triplet-state emission.

As a result, recent advances in ORTA luminescence have primarily focused on solid-state systems. Three main approaches are currently used to achieve ORTA in these systems: 1. Crystallization of luminescent small molecules: This helps to eliminate oxygen from the system and immobilizes molecular rotation, reducing the influence of non-radiative transitions [14,15,16,17,18,19,20,21,22,23,24,25,26]. 2. Polymer doping: This approach involves incorporating luminescent small molecules into a polymer matrix, utilizing a rigid polymer environment to exclude oxygen from the vicinity of the luminescent molecules [27,28,29,30,31,32,33,34]. 3. Co-doping with small molecules: This involves directly doping luminescent small molecules with another type of small molecule, which facilitates energy transfer between the two molecules, potentially leading to ORTA luminescence [35,36,37,38,39,40,41,42,43,44,45,46,47,48,49,50,51,52,53]. All three methods effectively suppress non-radiative transitions by reducing the quenching of triplet excitons, thereby achieving afterglow luminescence at room temperature. The latter two methods (polymer doping and co-doping) differ from the first (crystallization) by utilizing a host-guest approach. Doping offers several advantages over using pure luminescent materials. This enables a broader range of applications and can result in unexpected modes of energy transfer, as depicted in Figure 1, which can lead to exciting luminescent phenomena. For example, doping can create multiple luminescent systems simultaneously exhibiting TADF and RTP [32,44,45,46,47,48,49,50,51]; Exciplex formed by post-doping significantly prolongs the luminescence lifetime [46]; Two organic small molecules that would not exhibit room temperature afterglow (RTA) on their own can achieve RTA when doped together [54,55,56,57,58,59,60,61,62,63,64]. Recently, organic molecule-based ORTA luminescent systems, serving as doping hosts, have garnered attention from scientists due to their high tunability and compatibility with various guest molecules. This has spurred the development of a series of doping luminescent systems using various types of small molecules, which is furthering our understanding of ORTA mechanisms.

Solid-state systems offer a unique environment for manipulating luminescence. Various weak interactions, such as van der Waals forces, hydrogen bonds, π-π stacking, π-H interactions, and even halogen bonds, stabilize molecules in specific conformations within the crystal lattice. However, these conformations are not entirely rigid. External stimuli can trigger changes in a molecule’s conformation, which can then influence how molecules pack together in the crystal. This, in turn, fundamentally alters how electrons interact between molecules, leading to dramatic changes in their luminescent behavior [65,66,67,68,69]. Capitalizing on this concept, researchers have developed various solid-phase luminescence systems that utilize different physical stimuli to control the crystal packing and, consequently, modulate the light emission properties of small molecules. Common methods include recrystallization [69,70], solvent fumigation [65,66,67,68], and photoactivation [71,72,73]. These techniques highlight the responsiveness of solid-phase luminescence systems to various external triggers. Changes in physical conditions such as oxygen concentration [74,75], light intensity [71,72,73], and temperature directly manifest in the luminous color and lifetime [28,32,38,48]. This makes them highly promising candidates for sensor applications. This category of luminescent materials, also known as dynamically tunable ORTA materials, is highly compatible with future cutting-edge technologies due to its good reversibility, fast response, and tunable luminescence output. In this short review, we summarize some recent representative systems for achieving ORTA luminescence in the solid phase, including crystallization for afterglow, host-guest doping for creating multiple luminescence, and strategies for regulating their luminescence properties. We also discuss the key challenges encountered when studying solid-phase luminescent systems and propose potential research directions for the future.

## 2. Construction of Solid ORTA System

Solid-phase luminescence was first studied in crystals. Crystallization of organic small molecules creates a rigid oxygen-free environment, minimizing energy loss through non-radiative transitions. However, this method necessitates good crystallinity and restricts the emission spectra, limiting its applicability. Subsequently, strategies employing polymer matrices to provide a rigid oxygen-free environment have been developed. This approach extends its applicability to organic luminescent small molecules with poor crystallinity. Furthermore, polymer films offer greater flexibility compared to crystals, allowing for the creation of deformable luminescent films, thus expanding their utility. Additionally, doping luminescent small molecules (as hosts or matrices) with other small molecules enables afterglow luminescence. This method often results in multiple luminescence phenomena, including fluorescence, RTP, TADF, and mechanoluminescence (ML).

### 2.1. Crystallization

**Figure 2:**
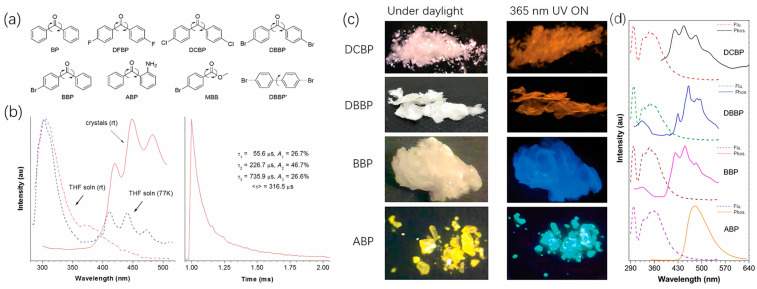
(**a**) Structures of BP and its derivatives; (**b**) PL spectra of BP in THF solution and crystalline state at room temperature and 77 K (left) and phosphorescence decay curve of BP crystals at room temperature. (**c**) Photographs of crystals of DCBP, DBBP, BBP, and ABP taken under normal laboratory lighting and 365-nm UV light illumination at room temperature. (**d**) the corresponding emission spectra of prompt fluorescence (dotted line) and phosphorescence (solid lines). Reproduced with permission from ref. [20] Copyright © 2024, American Chemical Society.

**Figure 3:**
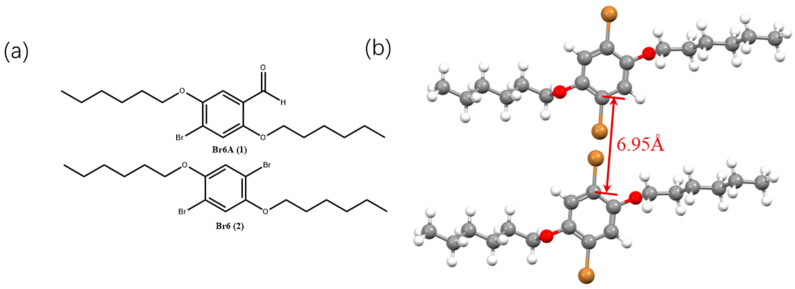
(**a**) Chemical structures of Br6A (1) and Br6 (2). (**b**) Diagram of Br6 crystal packing.

In 2010, Tang et al. pioneered the use of crystallization to induce efficient RTP, termed crystallization-induced phosphorescence (CIP) [20]. They utilized benzophenone (BP) as a core molecule and derived a series of small molecules exhibiting RTP (Figure 2a). The fluorescence emission peak of the BP solution was at 300 nm, while after recrystallization, a distinct phosphorescence emission peak at 450 nm was observed at room temperature, with a lifetime of 316 microseconds (Figure 2b). The phosphorescence lifetime was determined using a tri-exponential fit, indicating the existence of three emitting species. This could be attributed to diverse weak intermolecular interactions among BP molecules during crystallization, promoting the formation of various emissive aggregates, including dimers, trimers, and possibly larger oligomers, thereby giving rise to multiple emissive states. Such phenomena are frequently observed in crystalline room-temperature phosphorescence (RTP) [16,21]. Generally, the presence of heavy atoms can improve the efficiency of intersystem crossing, leading to stronger phosphorescence. However, the quantum yields of brominated derivatives, such as DBBP (12.0%), BBP (6.7%), ABP (8.6%), MBB (5.9%), and DBBP’ (13.9%), were lower than that of the native BP (15.9%). Bromine, with its larger atomic radius, profoundly influences the packing structure of crystals, indicating that the luminescence of crystals is primarily influenced by their stacking structure. In contrast, fluorinated DFBP exhibited a higher quantum yield of 39.7% due to fluorine forming weak interactions with C-H comparable to hydrogen bonds, enhancing crystal rigidity, and suppressing non-radiative relaxation processes, thus increasing the quantum yield. Figure 2c shows that DCBP and DBBP exhibit red-shifted emissions. The corresponding emission spectra reveal that, while their maximum emission peaks remain at around 450 nm, these compounds predominantly show new phosphorescence emission peaks in the 500–700 nm range compared to the blue emission of BP. Visually, their emissions appear distinctly orange. To exploit both heavy atom and crystallization effects simultaneously, Kim et al. designed molecules Br6 and Br6A with longer alkyl chains and heavy bromine atoms (Figure 3a) [14], increasing the bromine contact area in the crystals. While pure Br6 crystals displayed negligible luminescence, pure Br6A exhibited a phosphorescence lifetime of 5.4 ms and a quantum yield of 2.9%. Incorporating a 1% mass fraction of Br6 into Br6A to form a mixed crystal exhibits bright green luminescence under 365 nm excitation, corresponding to the emission spectrum within the 450–600 nm range, and significantly increases the quantum yield to 55%, resulting in a lifetime extension to 8.3 ms. Single-crystal data revealed a 0.01Å reduction in intermolecular distance with 1% Br6 in the Br6A crystal (Figure 3b). The addition of trace amounts of Br6 potentially facilitates triplet energy transfer, thereby significantly improving the quantum yield due to the heavy atom effect. This approach has led to the development of numerous crystallization-induced RTP systems, such as ImF and ImBr molecules exhibiting mechanoluminescent RTP [17], pyridine diphenyl sulfide molecules demonstrating proton-activated RTP [16], C-C4-Br molecules with red RTP [15], and DMACDPS molecules concurrently exhibiting TADF and RTP [21]. The appearance of these molecules has propelled the development of the field of crystallization-induced luminescence.

### 2.2. Polymer Host-Guest Doped System

**Figure 4:**
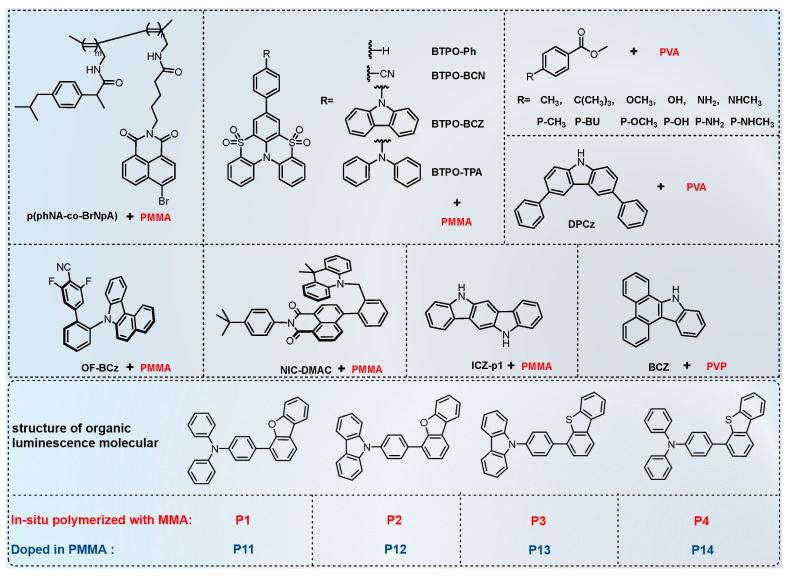
The chemical structures of the luminous small molecule guest used for polymers host doping system and in situ polymerization with MMA.

Bromonaphthalimide (BrNpA) has been incorporated onto helically chiral alkyne chains and doped into poly(methyl methacrylate) (PMMA) to create flexible p(phNA-co-BrNpA) films (Figure 4) capable of emitting circularly polarized room-temperature phosphorescence (CPL-RTP) [30]. While the chirally linked polyacetylene backbone promotes chirality, its weak rigidity limits the phosphorescence intensity. Doping the copolymer into PMMA creates a stiffer environment, hindering non-radiative deactivation and oxygen quenching. Photophysical studies revealed that the PMMA thin films exhibit a unique characteristic: the phosphorescence intensity gradually increases under continuous UV irradiation, peaking at 120 s, before decaying over 300 s. (Fluorescence intensity remains constant.) This behavior, observed across various copolymer ratios (5/5, 6/4, 7/3, 8/2), is attributed to the consumption of residual oxygen molecules by triplet sensitization under UV light (the rate of oxygen ingress into the polymer is lower than its consumption rate by the triplet excited state). Moreover, the helical polyacetylene chains effectively induce CPL in the phosphorescence. Notably, CPL-RTP can be activated by UV light and persists for 120 s after irradiation ceases. This property allows the films to be used as an erasable information storage medium. The authors successfully printed the copolymer onto various everyday materials (paper, wood, and glass) using a simple template printing technique, demonstrating its potential for anti-counterfeiting applications.

The sulfone-locked triphenylamine heteroaromatic core (BTPO) series molecules were doped into PMMA films (Figure 4), resulting in a high phosphorescence quantum yield (20%) and an ultralong phosphorescence lifetime (818 ms) [29]. Achieving both a long phosphorescence lifetime (τ_phos_) and high phosphorescence quantum yield (φ_phos_) in phosphorescent materials is challenging in general. In Equation (4), φ_phos_ is shown to be primarily influenced by φ_ISC_ and k_p_. Enhancing φ_phos_ directly involves designing molecules with a higher SOC constant to increase φ_ISC_. When φ_ISC_ shows minimal variation and k_p_ is sufficiently large, k_p_ becomes the dominant factor affecting φ_phos_. In such cases, φ_phos_ increases as k_p_ increases. Equation (5) indicates that the phosphorescence lifetime depends on k_p_, k_nr_, and k_q_ collectively and is inversely proportional to their sum. Thus, when k_nr_ and k_q_ have a minimal impact, a higher k_p_ results in a shorter lifetime. Achieving both high φ_phos_ and long τ_phos_ simultaneously necessitates high φ_ISC_ and low k_p_.

(4) $$\varphi_{phos}=\frac{\varphi_{ISC}\times k_{p}}{k_{p}+k_{nr}+k_{q}}$$

(5) $$\tau_{phos}=\frac{1}{k_{p}+k_{nr}+k_{q}}$$

where φ_ISC_ is the quantum yield of ISC, and k represents the transition rate related to each process: k_P_ is the phosphorescent decay rate, k_nr_ is the triplet non-radiative decay rate, and k_q_ is the overall quenching rate of T_1_ after integrating the concentration of quenchers.

Hence, molecules like BTPO were designed, incorporating heteroatoms (N, O, S) to narrow the energy gap for intersystem crossing and enhance the coupling constant. Furthermore, the D-A structure within BTPO significantly reduces the energy gap between its singlet and triplet states. However, due to its inherently planar rigid structure, the (π, π*) configuration of BTPO’s T_1_ orbital remains undisturbed. Through these strategic modifications, BTPO derivatives exhibit remarkable quantum yields and exceptionally prolonged RTP lifetimes in PMMA thin films. Photophysical experimental results reveal that doping PMMA thin films with 5 wt% BTPO derivatives yields φ_phos_ values of 11.7%, 20.2%, 15.6%, and 16.8% and corresponding τ_phos_ values of 542 ms, 818 ms, 380 ms, and 87 ms for BTPO-Ph, BTPO-BCN, BTPO-BCZ, and BTPO-TPA, respectively. Inkjet printing technology was utilized to apply PMMA organic solutions doped with 5 wt% BTPO derivatives for encrypted information applications.

To achieve both high φ_phos_ and an ultralong τ_phos_, OF-BCz was designed. It integrates polycyclic aromatic hydrocarbons (PAHs) as the electron donor and heavy-atom-containing difluorocyanobenzene at the N-terminus as the electron acceptor. This compound exhibits a τ_phos_ of 1.92 s and a φ_phos_ of 30% at 298 K in a PMMA film [31].

According to the El-Sayed rule, polycyclic aromatic hydrocarbons (PAHs) exhibit localized excited state ^3^π* (^3^LE) characteristics in the T_1_ state, granting them an ultralong RTP lifetime (τ_p_). However, due to their rigid structure, the efficiency of intersystem crossing is typically low. To enhance this efficiency, difluorodicyanobenzene containing multiple heavy atoms was tethered to the molecular skeleton, successfully increasing its quantum yield through localized excitation and greatly extending the RTP lifetime. In photolithography applications, OF-BCz can function as a photoinitiator for light-induced curing. Notably, residual OF-BCz molecules remain within the cured polymer, serving as an in situ quality control tool. By analyzing the fluorescence and phosphorescence properties, researchers can detect defects within photolithographic patterns.

**Figure 5:**
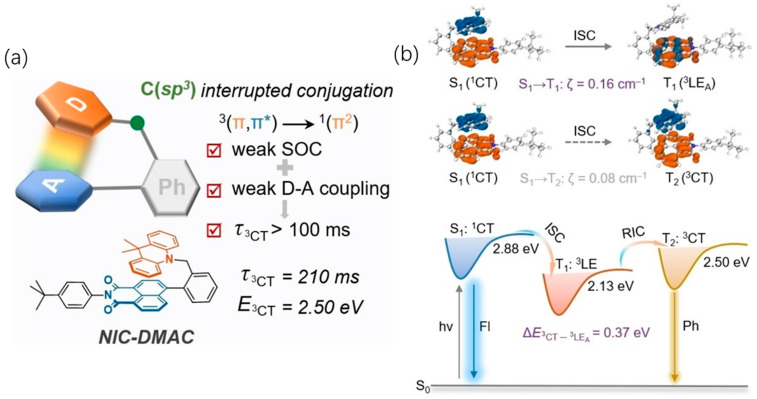
(**a**) Molecule design of NIC-DMAC; (**b**) natural transition orbitals of S_1_, T_1,,_ and T_2_ states for NIC-DMAC calculated in the gas phase by the TD-M062X/6–31g(d) method, blue for hole and orange for particle; SOCME (ζ) were calculated in the gas phase at the M062X/def2-tzvp level and the proposed mechanism for the ISC and RIC processes of NIC-DMAC. Reproduced with permission from ref. [23] Copyright © 2024 Wiley-VCH GmbH.

In another study, a naphthalimide-based luminophore was synthesized. This molecule features a 9,9-dimethylacridine (DMAC) electron donor unit connected to a naphthalimide acceptor unit via a benzyl group, creating a “sandwich-like” D-CH_2_(sp^3^)-A structure. This design promotes triplet excited state formation (^3^CT) within the PMMA films (Figure 4 NIC-DMAC). This innovative approach led to the development of the first single-molecule ultralong organic room-temperature phosphorescence (^3^CT-SMUOP) materials, boasting triplet lifetimes exceeding 100 milliseconds (Figure 5a) [23]. Computational studies reveal that, for the triplet state, both the “hole” and “particle” are localized on the naphthalene diimide molecule, facilitating a transition from the singlet S_1_ charge-transfer state to the T_1_ localized excitation (LE) state. However, this charge-transfer state does not emit light; instead, it undergoes reverse internal conversion (RIC) to revert to the ^3^CT state positioned at T_2_ for phosphorescence emission (Figure 5b). Capitalizing on its prolonged lifespan and high energy properties, they effectively engineered a photoinitiator, enabling dual patterning and implementing diverse anti-counterfeiting strategies.

For high-temperature phosphorescence, researchers have explored rigid 9H-dibenzo[a,c]carbazole (BCZ) molecules doped into a rigid polyvinyl pyrrolidone (PVP) polymer host (Figure 4) [28]. Both the planar structure of BCZ and the rigid nature of PVP minimize molecular motion, promoting the formation of a favorable electronic configuration (π, π*). This configuration reduces the rate of phosphorescence emission (k_p_) and extends the phosphorescence lifetime. Notably, this material exhibits a distinct afterglow lasting 1 s even at 433 K, making it suitable for personnel identification in low-visibility environments like fire rescue operations.

**Figure 6:**
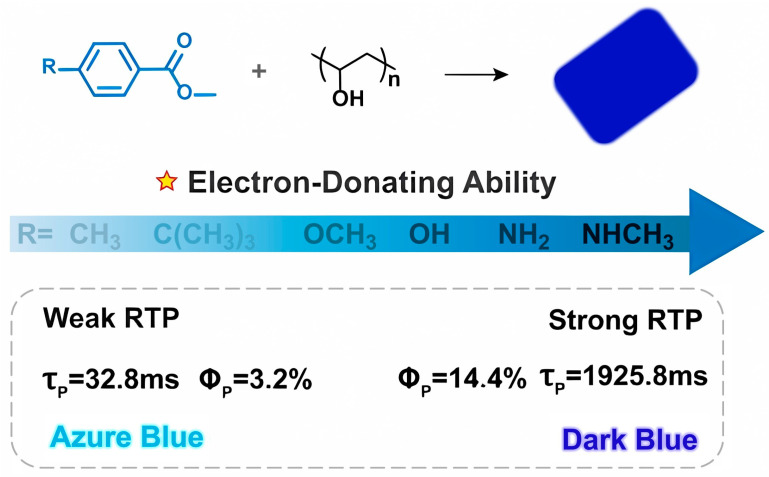
Construction strategy for efficient dark blue room-temperature phosphorescence with tunable lifetime. Reproduced with permission from ref. [33] Copyright © 2024 Wiley-VCH GmbH.

Another study explored the impact of guest-host interactions on phosphorescence properties. Traditionally, research has focused on the guest molecule’s structure when designing luminescent materials. However, this study highlights the importance of interactions between guest molecules and the polymer host (Figure 4 P series) [33]. In this work, researchers tuned the phosphorescence lifetime and intensity by modifying the guest molecule with substituents that form varying strengths of hydrogen bonds with the polyvinyl alcohol (PVA) host. Notably, modifying the guest molecule’s para position with different substituents led to a dramatic shift in phosphorescence: from weak, short-lived (32.8 ms) light blue emission to strong, ultralong-lived (1925.8 ms) deep blue emission, accompanied by a significant increase in quantum yield (Figure 6). This finding emphasizes the importance of considering guest-host interactions when designing efficient and long-lasting luminescent materials.

Furthermore, by utilizing the weak interactions between the OH groups in PVA and guest molecules, it is possible to form assemblies that exhibit ultralong-lived RTP. Among the series of luminescent molecules used for doping, PVA films doped with 7H-dibenzo[c,g]carbazole (DBCz) show the longest RTP lifetime (Figure 4), with a visible blue afterglow lasting up to 20 s at room temperature and a lifetime of 2044 ms [76]. Transparent PVA films prepared via this doping approach also possess good flexibility, allowing them to be folded into various shapes without compromising their luminescent performance.

**Figure 7:**
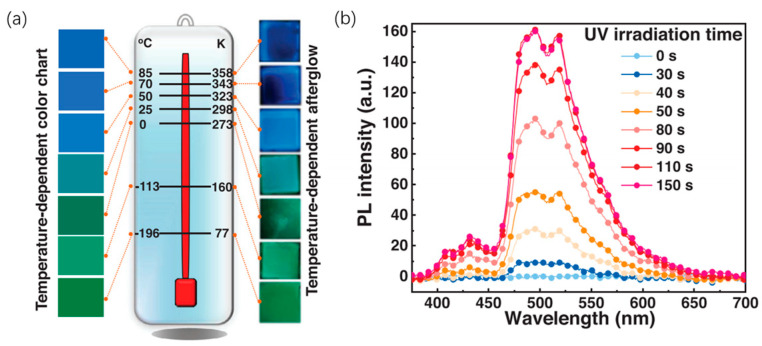
(**a**) Temperature-dependent afterglow of the ICZ-p1-0.5 wt.%-PMMA film and the corresponding temperature-dependent color chart for comparison temperature sensing. (**b**) Afterglow spectra at different UV light irradiation times of this film at 298 K in air. Reproduced with permission from ref. [32] Copyright © 2024 Wiley-VCH GmbH.

Indole (3,2-b)carbazole (ICZ-p1) (Figure 4) has recently been explored for its unique luminescent properties in PMMA thin films [32]. This material exhibits both TADF and RTP, allowing it to emit light under various conditions. Interestingly, the color of the emitted light can be tuned between blue and green depending on temperature and the amount of ICZ-p1 incorporated into the film (Figure 7). Moreover, ICZ-p1 demonstrates a fascinating characteristic: its luminescence intensity increases with prolonged light exposure (Figure 7b). This behavior can be attributed to the gradual consumption of oxygen molecules within the PMMA film by the excited state of the molecule. Taking advantage of its dual TADF and RTP emissions with distinct colors, researchers have designed a temperature sensor using ICZ-p1. At low temperatures, the sensor emits a green light due to the dominant TADF. In contrast, high temperatures favor RTP, leading to a blue emission (Figure 7a). Furthermore, ICZ-p1 shows promise for Organic Light-Emitting Diode (OLED) applications. Electroluminescent devices were fabricated using ICZ-p1 to achieve near-ultraviolet light emissions at low voltages. This paves the way for efficient and potentially low-power OLEDs.

Additionally, blending luminescent small molecules directly with MMA polymer monomers (Figure 4) and immobilizing them via in situ polymerization can achieve ultralong RTP [77]. In comparison to mere doping of luminescent small molecules into PMMA (where P11 to P14 exhibit τ_phos_ of 0.29 ms, 0.32 ms, 0.61 ms, and 0.31 ms, respectively), their phosphorescence lifetimes are significantly extended (P1 to P4 exhibit τ_phos_ of 0.77 s, 1.51 s, 0.25 s, and 0.26 s, respectively). Moreover, their phosphorescence quantum yields also demonstrate an improvement (P1 to P4 show φ_phos_ values of 3.80%, 1.07%, 0.43%, and 2.90%, respectively; P11 to P14 show φ_phos_ values of 1.76%, 1.06%, 0.26%, and 0.99%, respectively). This suggests that the in situ polymerization approach effectively limits molecular mobility and reduces oxygen quenching of triplet excited states, offering a novel approach for designing room-temperature phosphorescent materials.

However, polymer films like PMMA have limitations. Their long polymer chains can be sensitive to harsh environments with strong acidity, alkalinity, or moisture. This necessitates the development of pure organic small molecule systems like ICZ-p1 for applications requiring greater environmental stability.

### 2.3. Multiple ORTA Luminescence of Organic Molecules

**Figure 8:**
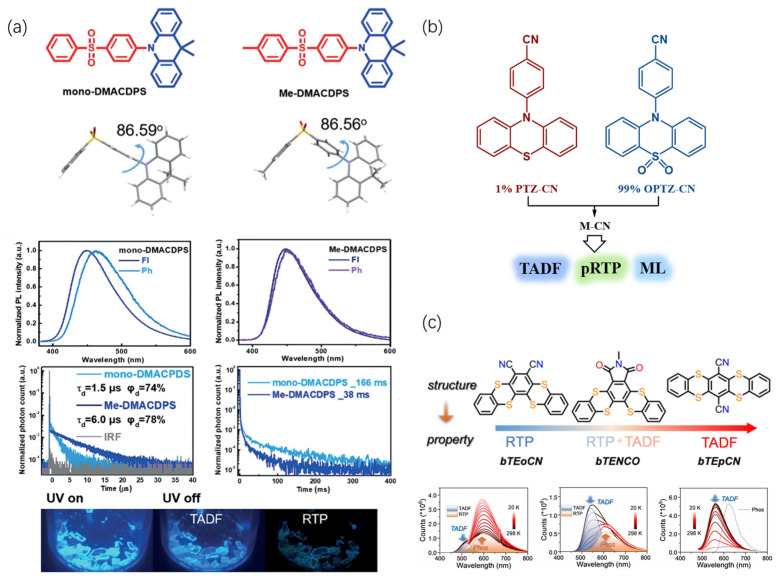
(**a**) Molecular and single-crystal structures of mono-DMACDPS and Me-DMACDPS; Normalized PL spectra of mono-DMACDPS and Me-DMACDPS in the crystal at 300 K. Transient photoluminescence spectra of mono-DMACDPS and Me-DMACDPS in the crystal: image of mono-DMACDPS crystal with 365 nm lamp on and off. Reproduced with permission from ref. [21] Copyright © 2024 Wiley-VCH Verlag GmbH & Co. KGaA, Weinheim (**b**) Exciplex-induced TADF, persistent RTP, and ML in a doping system with OPTZ-CN as the host and PTZ-CN as the guest. (**c**) Molecular structures and temperature-dependent steady-state PL spectra of bTEoCN, bTENCo, and bTEpCN. Reproduced with permission from ref. [41] Copyright © 2024 Wiley-VCH GmbH.

Energy or electron donor and acceptor can form complexes in the excited state in the solid phase, leading to multiple luminescences. Co-crystallization is a powerful tool for obtaining materials with multiple luminescence properties. For example, covalently linking the electron-donating molecule DMAC with the electron-accepting molecule Diphenyl sulfone (DPS) resulted in crystals exhibiting a combination of blue RTP, ML, and TADF [21]. As depicted in Figure 8a, the nearly perpendicular arrangement between the donor and acceptor in the crystal structure minimizes the energy gap between the singlet and triplet states (0.014 eV). This facilitates efficient reverse intersystem crossing (RISC), leading to TADF emission. Interestingly, the TADF and RTP peaks in the emission spectrum are very similar in color (10 nm difference), but their lifetimes are distinct. TADF has a lifetime of either 1.5 μs or 6.0 μs, while RTP lifetimes range from 38 ms to 166 ms. This confirms the multiple luminescence behavior. This corroborates the multiple ORTA luminescence behavior. Additionally, DMACDPS molecules in the crystals form dimers, endowing them with charge separation properties and exhibiting ML. Conversely, the methyl-modified Me-DMACDPS molecules disrupt molecular aggregation, eliminating the ML characteristics.

Another approach to achieve multiple luminescence involves using small molecule doping systems. Researchers have designed and synthesized a series of molecules based on a thianthrenequinone core (Figure 8c) [41]. By varying the positions of the cyano and other substituents, they obtained molecules with diverse luminescent properties. For example, pTEoCN exhibits only RTP afterglow, bTENCO exhibits both RTP and TADF, and bTEpCN exhibits only TADF afterglow. Doping these molecules into a host material (CBP) allows them to fine-tune the overall luminescence properties. Importantly, doping can also achieve ultralong phosphorescence and TADF through the formation of an exciplex.

Phenothiazine was utilized as the scaffold for triplet luminescence, and its N-terminus was modified with para-cyanobenzene, oxidizing sulfur to yield two luminescent molecules, PTZ-CN and OPTZ-CN (Figure 8b). OPTZ-CN served as the host, with 1% PTZ-CN guest doping to form a mixed crystal (M-CN) [46]. This doping significantly enhanced the phosphorescence quantum yield from a maximum of 16% to 61%. The resulting material displayed a combination of TADF, persistent RTP, and ML, with an impressive RTP afterglow lasting for up to 240 *s*. Analysis suggests that an exciplex might form between the two molecules, promoting triplet energy exchange and improving light emission efficiency.

Carbazoles [38,39,42,43,47], phenothiazines [44,46,78], and thianthrene [41] are popular choices for creating materials with multiple luminescent properties, including both TADF and RTP. These molecules are favored because they efficiently utilize light energy and possess suitable electronic structures for triplet excited states. By incorporating these molecules into different systems, researchers have developed a variety of materials with unique combinations of TADF and RTP. This progress has significantly advanced the field of ORTA luminescence.

## 3. Regulation of Solid ORTA

Various methods have been proposed for modulating luminescence in solid-state systems. These methods include inducing different crystal forms from the same organic molecule during crystallization, which can adjust the emission intensity of the RTP or alter its afterglow emission type [65,66,67,68,69]. Furthermore, solid-state afterglow luminescence can be controlled using various physical stimuli such as solvent vaporization [65,66,67,68], light stimulation [71,72,73], oxygen concentration [74,75], and mechanical grinding [17,46,79,80]. Moreover, owing to the stability of triplet states in this type of luminescence, it holds promise for utilization in upconversion luminescent systems [81].

**Figure 9:**
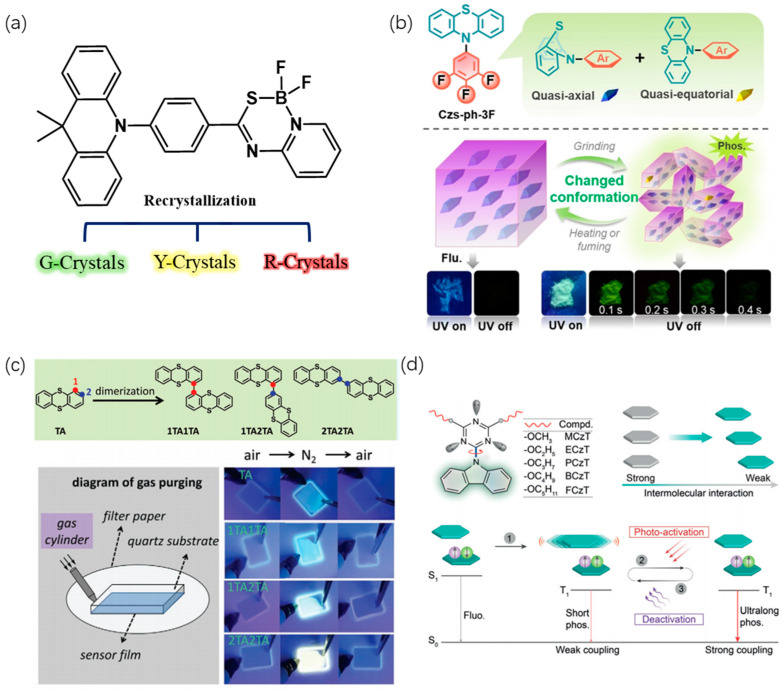
(**a**) Molecular structure and single crystals of the G-crystal Y-crystal, and R-crystal of DMAC-PSBF2. (**b**) The molecular structure and two possible conformations of CzS-ph-3F and a proposed diagram for the stimulus-responsive RTP effect based on conformational changes and photographs of Czs-ph-3F taken before and after turning off 365 nm UV irradiation under ambient conditions. Reproduced with permission from ref. [79] Copyright © 2024 Wiley-VCH GmbH. (**c**) Molecular structures of TA analogs and images of TA analogs:PMMA films under 254 nm hand lamp, before and after elimination of oxygen by nitrogen purging, respectively, the numbers 1 and 2 in molecule TA means the different reactive sites). Reproduced with permission from ref. [74] Copyright © 2024 Wiley-VCH GmbH. (**d**) Rational design of molecular rotors, molecular packing modes in crystals, and proposed mechanism for dynamic ultralong organic phosphorescence (step 1: intersystem crossing; step 2: photo-activation, and step 3: thermally deactivation). Reproduced with permission from ref. [71] Copyright © 2024 Wiley-VCH Verlag GmbH & Co. KGaA, Weinheim.

**Figure 10:**
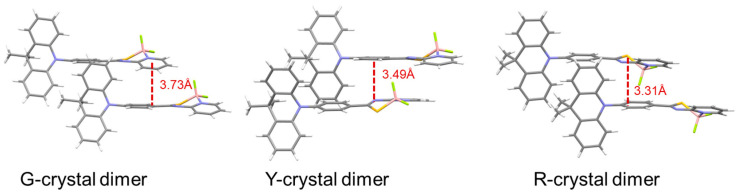
The adjacent dimers in single crystals of DMAC-PSBF2 (G-crystal, Y-crystal, and R-crystal).

To regulate solid-state luminescence, DMAC-PSBF2 was devised, which comprises DMAC as the electron donor and N,S-thioamide difluoroboron (PSBF2) as the electron acceptor, yielding three distinct crystal structures. These luminescent molecules display three luminescent modes: green TADF, yellow RTP, and red TADF, corresponding to the three crystal forms denoted as G, Y, and R-crystals (Figure 9a). The three crystals were crystallized in three different mixed solvents: G-crystal (DCM:n-hexane = 1:1), Y-crystal (MeOH:n-hexane = 1:1), and R-crystal (toluene:n-hexane= 1:2). Analysis of the three single-crystal structures reveals variations in the dihedral angle formed by the benzene ring of the DMAC fragment. Specifically, the dihedral angle in the R-crystal is 10°, indicating a nearly planar conformation and maximum conjugation. In contrast, the DMAC segments in the G and Y-crystals exhibit larger dihedral angles of 39° and 43°, respectively. Furthermore, when considering intermolecular distances (measured from the π-plane of one molecule to the nearest atom of another), the R-crystal shows the shortest distance at 3.31 Å (S⋯π), whereas the distances in the other two crystals are 3.73 Å (C–H⋯π) and 3.49 Å (N⋯π), respectively (Figure 10). This suggests a significant overlap of electron clouds between dimers in the R-crystal, resulting in a red-shifted TADF emission. The Y-crystal features notable C–H⋯F (2.40 Å, 2.41 Å, and 2.53 Å), C–H⋯π (2.68 Å, 2.79 Å, and 2.88 Å), and S⋯π (3.46 Å) interactions, forming a three-dimensional framework. Consequently, this crystal exhibits the highest density of 1.439 g cm^−3^ (compared to 1.379 g cm^−3^ for the G-crystal and 1.365 g cm^−3^ for the R-crystal), creating a dense structure that isolates oxygen and restricts molecular motion, thus demonstrating RTP characteristics. While the quantum yield can reach up to 77% (Y-Crystals), the lifetimes are relatively short, with the longest being 3.1 μs (G-Crystals) [69].

**Figure 11:**
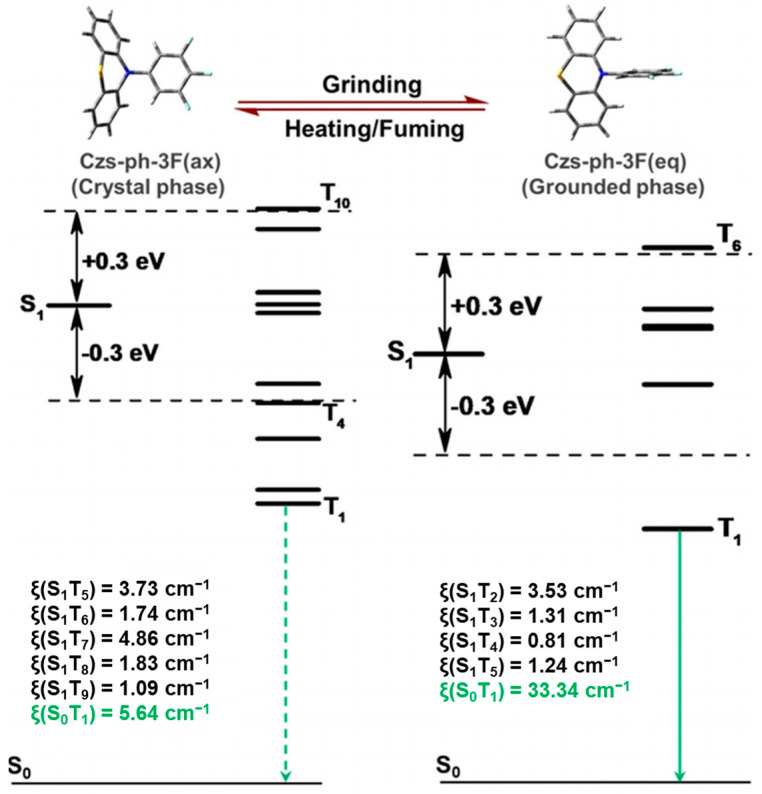
Calculated SOC constants (ξ) between the S_0_/S_1_ and T_n_ (Δ*E*_S1Tn_ < 0.3 eV) for Czs-ph-3F(ax.) and Czs-ph-3F(eq.). Reproduced with permission from ref. [79] Copyright © 2024 Wiley-VCH GmbH.

Additionally, modifying the nitrogen end of phenothiazine with trifluorotoluene enables the molecule (Csz-ph-3F) to undergo a reversible transformation between equatorial (eq.) and axial (ax.) conformations (Figure 9b) under conditions such as heating, solvent vaporization, and grinding in the solid state [79]. PXRD test results revealed no significant alterations in the characteristic solid-state peaks following grinding, vaporization, or heating, indicating that physical stimuli do not affect the stacking mode of the solid; instead, they induce conformational changes in the molecule. From DFT calculations, it is evident that grinding-induced conformational changes significantly alter the molecular charge distribution (Figure 11). In the post-grinding eq. conformation, there is notable intramolecular charge transfer, leading to enhanced spin-orbit coupling between singlet and triplet states, particularly evidenced by an SOC constant of 33.34 cm^−1^ (ax. is 5.64 cm^−1^). This suggests that the eq. conformation favors phosphorescence emission. This phenomenon explains the occurrence of room-temperature phosphorescence (RTP) after grinding, attributed to the transformation of molecular conformation from ax. to eq. with a higher SOC constant, thereby facilitating RTP emission. The authors suggested the potential application of this molecule in thermal-sensitive printing.

**Figure 12:**
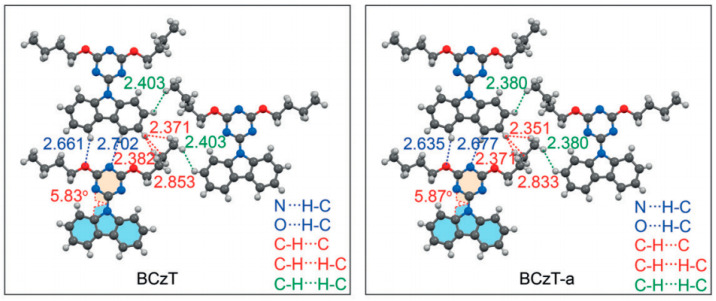
Intermolecular interactions and twisting angles of BCzT in a single crystal before and after photoactivation (BCzT-a) at 100 K. Reproduced with permission from ref. [71] Copyright © 2024 Wiley-VCH Verlag GmbH & Co. KGaA, Weinheim.

Furthermore, RTP is known to be sensitive to oxygen. Based on this, scientists have synthesized three oxygen-sensitive RTP materials by covalently bonding anthracenes at different positions [74]. They observed a significant impact of the anthracene substitution position on the phosphorescence quantum yield, which increased from 7.9% for 1TA1TA to 40.7% for 2TA2TA (Figure 9c). Upon doping the PMMA films, the room-temperature phosphorescence signal was detectable only under N_2_ flow. Oxygen concentration tests demonstrated the luminescent system’s high sensitivity to oxygen, with RTP extinguishing at just 1.7% oxygen concentration, rendering it suitable for precision oxygen concentration sensor manufacturing. Additionally, to enable control of the luminescence duration through light exposure, a series of luminescent molecules featuring a triazine core, carbazole unit, and alkoxy chain was devised and synthesized [71]. It was found that PCzT, BCzT, and FCzT (Figure 9d) displayed pronounced photoactivated phosphorescence. Upon extended light exposure, the solid-state configuration of these molecules experienced a heightened release of triplet exciton energy, facilitated by rapid molecular conformational vibrations, leading to photoactivated phosphorescence. Analysis of the crystal structure before and after photoactivation reveals that the interaction distance between adjacent molecules on the same plane decreases following photoactivation. Specifically, the distances between carbazole and triazine groups decrease from 2.661 Å and 2.702 Å to 2.635 Å and 2.677 Å, and between carbazole and butoxy groups from 2.382 Å and 2.853 Å to 2.351 Å and 2.833 Å. The interaction distance between adjacent butoxy substituents on the same plane decreases from 2.343 Å to 2.293 Å (Figure 12). This reduction in intermolecular distances significantly strengthens intermolecular interactions, constraining molecular free motion, thereby reducing the proportion of non-radiative triplet exciton dissipation and activating RTP. This photoactivated phosphorescence can transition back to a non-emissive state through thermal relaxation or heating, showing significant implications for the development of anti-counterfeiting materials.

## 4. Summary and Outlook

Research on solid-state luminescent systems is still in its early stages. Luminescence, being a phenomenon most intuitively observed by the human eye, theoretically represents the most efficient sensor when manipulated through various physical stimuli, given the rapid propagation of light. Therefore, it is crucial to thoroughly understand the luminescence mechanism in solid-state systems. Currently, explanations for solid-state luminescence primarily focus on differences in molecular spacing and molecular folding angles within the crystal stacking structures. However, computational chemistry often struggles to directly calculate using stacking structures due to the complexities of intermolecular interactions and arrangements. The results obtained from such calculations often lack precision. Consequently, most discussions on excited-state electronic transitions are based on single-molecule structures or dimer structures, which may not accurately reflect the actual stacking structures and fail to provide a precise explanation for the fundamental reasons behind this type of luminescence. Therefore, it is challenging to develop a universal theory to explain solid-state luminescence.

Additionally, solid-state luminescence not only requires the molecules themselves to exhibit good luminescent properties but also relies heavily on the molecular stacking structures within the crystal. Predicting crystal stacking structures directly from molecular structures is difficult, and information regarding stacking arrangements can only be obtained through X-ray diffraction after obtaining crystals. Thus, the approaches to designing luminescent solid-state systems are rather passive. Most of the time, scientists can only interpret why such luminescent phenomena occur based on experimental observations rather than directly predicting luminescence through their stacking structures. However, the application scenarios of solid-state luminescence systems are broader than those of liquid-phase systems, and they exhibit more diverse and unique properties. Therefore, a systematic investigation of the principles of solid-state luminescence is necessary for our subsequent research and design endeavors. The current research on luminescent solid-state systems has only scratched the surface, and there is much more to explore.

## Data Availability

Not applicable.
